# Plasma transferrin and hemopexin are associated with altered Aβ uptake and cognitive decline in Alzheimer’s disease pathology

**DOI:** 10.1186/s13195-020-00634-1

**Published:** 2020-06-09

**Authors:** Azhaar Ashraf, Nicholas J. Ashton, Pratishtha Chatterjee, Kathryn Goozee, Kaikai Shen, Jurgen Fripp, David Ames, Christopher Rowe, Colin L. Masters, Victor Villemagne, Abdul Hye, Ralph N. Martins, Po-Wah So, AIBL

**Affiliations:** 1 Department of Neuroimaging, Institute of Psychiatry, Psychology and Neuroscience, Maurice Wohl Clinical Neuroscience Institute, King’s College London, 5, Cutcombe Road, Denmark Hill Campus, London, SE5 9RX UK; 2 Institute of Psychiatry, Psychology and Neuroscience, Maurice Wohl Institute Clinical Neuroscience Institute, King’s College London, London, UK; 3 NIHR Biomedical Research Centre for Mental Health and Biomedical Research Unit for Dementia at South London and Maudsley NHS Foundation, London, UK; 4 Department of Psychiatry and Neurochemistry, The Sahlgrenska Academy at the University of Gothenburg, Mölndal, Sweden; 5 Wallenberg Centre for Molecular and Translational Medicine, University of Gothenburg, Gothenburg, Sweden; 6 School of Medical Sciences, Edith Cowan University, 270, Joondalup, WA 6027 Australia; 7 KaRa Institute of Neurological Diseases, Macquarie Park, NSW Australia; 8 Department of Biomedical Sciences, Macquarie University, North Ryde, NSW 2109 Australia; 9 Clinical Research Department, Anglicare, Sydney, NSW Australia; 10 School of Psychiatry and Clinical Neurosciences, University of Western Australia, Crawley, WA Australia; 11 Australian E-Health Research Centre, CSIRO, Brisbane, Australia; 12 Rapiscan Systems, St Leonards, Sydney, Australia; 13 Academic Unit for Psychiatry of Old Age, St. George’s Hospital, University of Melbourne, Victoria, VIC Australia; 14 National Ageing Research Institute, Parkville, VIC Australia; 15 Department of Molecular Imaging and Therapy, Austin Health, Heidelberg, VIC Australia; 16 The Florey Institute, University of Melbourne, Parkville, VIC Australia

**Keywords:** Alzheimer’s disease, Cognitively normal, Cognitive impairment, Heme, Hemoglobin, Iron, Mild cognitive impairment, Proteomics, Transferrin

## Abstract

**Background:**

Heme and iron homeostasis is perturbed in Alzheimer’s disease (AD); therefore, the aim of the study was to examine the levels and association of heme with iron-binding plasma proteins in cognitively normal (CN), mild cognitive impairment (MCI), and AD individuals from the Australian Imaging, Biomarker and Lifestyle Flagship Study of Ageing (AIBL) and Kerr Anglican Retirement Village Initiative in Ageing Health (KARVIAH) cohorts.

**Methods:**

Non-targeted proteomic analysis by high-resolution mass spectrometry was performed to quantify relative protein abundances in plasma samples from 144 CN individuals from the AIBL and 94 CN from KARVIAH cohorts and 21 MCI and 25 AD from AIBL cohort. ANCOVA models were utilized to assess the differences in plasma proteins implicated in heme/iron metabolism, while multiple regression modeling (and partial correlation) was performed to examine the association between heme and iron proteins, structural neuroimaging, and cognitive measures.

**Results:**

Of the plasma proteins implicated in iron and heme metabolism, hemoglobin subunit β (*p* = 0.001) was significantly increased in AD compared to CN individuals. Multiple regression modeling adjusted for age, sex, APOEε4 genotype, and disease status in the AIBL cohort revealed lower levels of transferrin but higher levels of hemopexin associated with augmented brain amyloid deposition. Meanwhile, transferrin was positively associated with hippocampal volume and MMSE performance, and hemopexin was negatively associated with CDR scores. Partial correlation analysis revealed lack of significant associations between heme/iron proteins in the CN individuals progressing to cognitive impairment.

**Conclusions:**

In conclusion, heme and iron dyshomeostasis appears to be a feature of AD. The causal relationship between heme/iron metabolism and AD warrants further investigation.

## Background

Alzheimer’s disease (AD) is the most common cause of dementia in the elderly population, with an estimated prevalence of 115.4 million cases by 2050 [1]. AD is a heterogenous disorder characterized clinically by apraxia, aphasia, and agnosia and neuropathologically by the presence of β-amyloid plaques and hyperphosphorylated tau tangles [2, 3]. Regional measures of hippocampal atrophy have been identified as strong predictors of progression to AD [4]. Emerging evidence suggests that brain and blood iron homeostasis is perturbed in AD, with studies showing decreased peripheral iron (anemia) and lower hemoglobin levels [5–11]. Anemia is a prevalent condition in the elderly and is associated with an increased risk of acquiring AD [7, 8, 12, 13].

Evidence suggests that hemoglobin (Hb), a group of iron-containing heme proteins, is not exclusively restricted to the blood but likely play a role in pathophysiology of the brain and neurodegenerative diseases [14]. Hb acts likely as an oxygen storage molecule to counteract hypoxic effects on neurons, which have high obligate demand for sustained energy [14, 15]. Hb is expressed throughout the brain including the striatum, corpus callosum, and brainstem [14, 15]. High levels of Hb components are evident in brains of older humans and rats [16], associated with impaired learning and memory [17]. Hb interacts with β-amyloid (Aß) and co-localizes with Aß plaques in AD post-mortem brains [18, 19]. Importantly, Hb levels were augmented in amyloid pathology-associated brain regions—inferior temporal gyrus, cerebral parietal gray matter, and parietal white matter [19]. The association between brain region-specific Hb levels and Aβ pathology in AD patients suggests a link between Hb expression and AD pathogenesis.

Although research evaluating the differences between individual subunits of Hb remains sparse, there is evidence that specific subunits have varying properties and functions. Studies of blood from healthy humans show that Hbα subunits efficiently scavenge hydrogen peroxide to prevent oxidative degradation of Hb [20]. Hbα-positive neurons, juxtaposing Hbα-negative neurons, have higher oxygenation potential in hypoxic areas in the mouse brain under normoxic and hypoxic conditions [21]. Interestingly, Hbα levels were predominantly decreased in brain synaptosomes in a transgenic rat model of AD [22]. Meanwhile, Hbβ-enriched neurons are frequent in internal cortical layers IV–VI relative to external layers I–III; most of these neurons had pyramidal morphology with large size, apical dendrites, and long axons [23]. It was found that overexpression of Hbβ but not Hbα epigenetically increased histone H3 methylation to sequester oxygen from histone demethylases [23]. Although the α and β subunits of Hb function as a heterotetramer in erythrocytes, these proteins have putatively evolved specialized functions and may act independent of one another in selected cells. This proposition is further supported by the expression of Hbα in vascular endothelial cells where it regulates nitric oxide signaling, independent of Hbβ [24].

In neurons laden with hyperphosphorylated tau deposits, appreciable decreases in Hb subunits α and β were evident in the frontal cortex and hippocampus in AD [25]. Analysis of samples from the CSF showed higher Hbα and Hbβ which could differentiate between AD converters from non-converters in individuals with mild cognitive impairment (MCI) [26]. However, Hb subunits did not yield significant differences between CN, MCI, and AD. This suggests that Hb subunits may show an initial increase in response to AD pathology but due to ongoing disease, Hb may leak from degenerating neurons into CSF and explain the augmented CSF levels in MCI converting to AD. Note that a stringent protocol was implemented to avoid blood contamination to ensure removal of plasma proteins from CSF samples to assure robustness of results [26]. The role of another Hb subunit, Hbδ, in aging and disease has not been investigated. Hbδ genes show homology to Hbβ [15]; hence, it can be hypothesized to follow similar changes to Hbβ in the context of AD.

Hb oxidation liberates free heme, a source of redox-active iron, which produces reactive oxygen species through Fenton reaction, inducing lipid peroxidation [27]. This toxicity is modulated by hemopexin (HPX) and haptoglobin, proteins which bind heme with high affinity to maintain iron homeostasis through recycling of heme iron [28, 29]. Proteomics studies demonstrated increased plasma and CSF HPX in AD patients compared to cognitively normal (CN) subjects, suggesting impaired compensation in neurodegeneration [30, 31]. It appears that surplus contribution to heme by breakdown of Hb may overwhelm the capacity of the heme scavenging system in AD [28, 32–34].

Systemic deficits in iron trafficking can contribute to morbidity associated with AD, since iron acts as a catalyst for a plethora of metabolic processes, including heme formation, neurotransmitter synthesis, and axonal myelination [7, 8]. Deficiency of iron loading onto its major transporter protein, transferrin, was observed in AD [8], which suggests further examination of peripheral iron/heme metabolism.

Since AD represents a global disease burden, plasma proteins have been extensively associated with progression of the disease [35]. The present study investigated the levels of plasma proteins involved in heme and iron homeostasis in cognitively normal (CN), MCI, and AD individuals. The uniqueness of the study was that it identified CN individuals who either remained stable or showed progression to cognitive impairment and examined retrospectively their baseline plasma proteins and association to AD markers. The baseline expression levels of plasma hemoglobin (Hb) subunits (α, β, and δ) and their association to AD markers have not been previously documented, highlighting the importance of the present study. Based on the evidence reviewed, we hypothesize that Hbβ would be associated with higher Aβ deposition, hippocampal atrophy, and cognitive decline. We also predict that higher levels of Hbα, hemopexin, and transferrin will be associated with lower Aβ deposition, improved hippocampal volumes, and improved cognitive performance.

## Methods

### Participants

The dataset comprised 144 CN, 21 MCI, and 25 AD individuals derived from the Australian Imaging, Biomarker and Lifestyle Flagship Study of Ageing (AIBL [11, 36, 37]) and 94 CN from Kerr Anglican Retirement Village Initiative in Ageing Health (KARVIAH) cohort [38]. The AIBL cohort underwent baseline Aβ PET imaging (^11^C-PIB) [37], while KARVIAH cohort had baseline ^18^F-FBB (florbetaben) amyloid PET imaging [11]. Only the AIBL individuals included in the study received magnetic resonance imaging (MRI) using the ADNI three-dimensional magnetization prepared rapid gradient echo (MPRAGE) sequence, with the following parameters: 1 × 1 mm in-plane resolution, 1.2 mm slice thickness, field of view 240 × 256, 160 slices, repetition time (TR) = 2300 ms, echo time (TE) = 2.98 ms, inversion time (TI) = 900 ms, and flip angle 9°. T2-weighted fast spin echo (FSE) and fluid attenuation inversion recovery (FLAIR) sequences were also applied [37]. MRI-based measures of hippocampal, gray matter, and white matter volumes were derived.

AIBL and KARVIAH subjects had mini-mental state examination (MMSE) [11, 37], while clinical dementia rating scale (CDR) and sum of boxes (CDR SB) were computed for the AIBL cohort [37].

### Inclusion and exclusion criteria for KARVIAH

Participants with good health and no history of significant cerebral vascular disease, fluent in English, with adequate vision and hearing to enable testing, and with normal general cognitive function as determined by Montreal Cognitive Assessment (MoCA) [39] scores greater than or equal to 26 were included. The exclusion criteria included diagnosis of dementia based on the revised criteria from the National Institute on Aging - Alzheimer’s Association, presence of acute functional psychiatric disorder (including lifetime history of schizophrenia or bipolar disorder), history of stroke, and presenting with depression based on the Depression, Anxiety, Stress Scales (DASS) and uncontrolled hypertension (systolic BP > 170 or diastolic BP > 100).

### Inclusion and exclusion criteria for AIBL

CN individuals were free of cognitive impairment, while MCI was defined as a clinical syndrome characterized by reduced cognitive performance (often involving memory), representing a risk state for development of frank AD [40, 41]. The diagnosis of AD was made according to the National Institute of Neurological and Communicative Disorders and Stroke - Alzheimer’s Disease and Related Disorders Association (NINCDS-ADRDA) criteria [42]. Allocation of individuals to CN, MCI, or AD was undertaken by a clinical review panel (see below). When individuals presented with an existing diagnosis of MCI or AD, the diagnosis was established by a clinical review panel to ensure that diagnoses were made according to internationally agreed criteria. Potential participants were excluded if they had a history of non-AD dementia, schizophrenia, bipolar disorder, significant depression (GDS score > 5/15), Parkinson’s disease, cancer within last 2 years, stroke, or uncontrolled diabetes.

Dementia severity was rated for all participants with CDR = 0, 0.5, 1, 2, or 3 when dementia is absent, questionable, mild, moderate, or severe, respectively [43]. Since six domain scores ranging from 0 to 3 are generated for CDR, a sum of boxes score is also calculated ranging from 0 to 18.

Individuals that participated as CN at baseline in the AIBL trial were discussed by a clinical review panel (comprising neurologists, old age psychiatrists, geriatricians, and neuropsychologists). For individuals with an MMSE score < 28, failure on the logical memory test (as per ADNI guidelines, http://adni.loni.usc.edu/), and/or a CDR score ≥ 0.5 [36], a consensus diagnosis was assigned based on diagnostic criteria DSM-IV [44] and ICD-10 [45] and whether the subject violated the exclusion criteria. CN individuals who progressed to fulfill MCI diagnosis according to established Winblad et al. [41] and Petersen et al. criteria [40] had to also have impairment on two or more cognitive tests at a level of at least 1.5 standard deviation below age-adjusted mean, in addition to have reported memory difficulties. Individuals were assigned a diagnosis of probable AD if they reported deficits in multiple cognitive domains and impairment in activities of daily living sufficient to satisfy NINCDS-ADRDA [42], DSM-IV [44], and ICD-10 [45] criteria.

### Mass spectrometry analysis

The methodology used in this study has been previously described [38]. In brief, AIBL and KARVIAH plasma samples were immuno-depleted of albumin and IgG, and then enzymatically digested prior to tandem mass tag (TMT) labeling. The resulting peptides were separated into 24-fractions by an OFFGEL Fractionator (Agilent Technologies) and individually analyzed by a Linear Trap Quadrupole (LTQ) Orbitrap Velos Pro (Thermo Fisher Scientific), enabling chromatographic separation and mass spectra acquisition.

### Statistical analyses

Proteins involved in iron and heme metabolism were selected from a panel of plasma proteins [38] and analyzed, which included transferrin, ceruloplasmin, hemopexin, haptoglobin, haptoglobin-related protein (HPR), Hb subunits (α, β, δ), cluster of differentiation 163 (CD163), and low-density lipoprotein receptor-related protein (LRP1). ANCOVA models were used to compare the baseline levels of plasma proteins, neuroimaging, and cognition measures between the diagnostic groups (CN, MCI, and AD), adjusted for age, sex, APOEε4 genotype, and disease status. Multiple regression models were utilized to study the association of plasma proteins with neuroimaging measures and cognitive assessments, where minimal models were obtained using backward elimination.

Subsequently, the CN subset from the AIBL cohort was dichotomized into individuals that progressed to show symptoms of cognitive impairment and individuals that remained stable, 6 years later. ANCOVA and linear regression analysis was performed as described above but the models were adjusted for age, sex, APOEε4 genotype, and conversion status. To delineate the association of proteins involved in iron and heme metabolism, partial correlation analysis was executed, adjusting for age, sex, and APOEε4 genotype in CN individuals who remained stable and in individuals progressing to cognitive impairment.

To correct for multiple comparisons, we used an adaptive linear set-up procedure proposed by Benjamini et al. [46] to control the false discovery rate (FDR) at 5% across all the analyses. An FDR-corrected *p* value ≤ 0.0167 was considered significant. All statistical analyses were performed using IBM SPSS Statistics 24 and GraphPad Prism 8.4.2.

## Results

### Baseline levels of plasma heme and iron proteins in CN, MCI, and AD

The baseline demographic and clinical characteristics of the different diagnostic groups are presented in Table 1. AD subjects exhibited increased brain amyloid deposition compared to CN and MCI, while MCI had higher brain amyloid deposition than CN (*p* = 3.224 × 10^−20^). The right (*p* = 0.008) and left hippocampal (*p* = 3.600 × 10^−5^) volumes were found to be markedly reduced in AD relative to CN. Individuals with AD had lower MMSE scores and increased CDR and sum of boxes compared to CN and MCI, while the MCI group performed worse on MMSE (*p* = 9.453 × 10^−54^) and had higher CDR (*p* = 9.139 × 10^−56^) and sum of boxes (*p* = 3.384 × 10^−73^) compared to CN.

**Table 1:** Baseline demographic and clinical characteristics of CN, MCI, and AD subjects

		CN	MCI	AD	*p* value
*n*	–	144 AIBL, 94 KARVIAH	21	25	NA
Age	Years (s.d.)	75 (7.362)	75 (6.226)	71 (9.072)	0.170
Female	*n* (%)	105 (44%)	9 (43%)	13 (52%)	0.743
APOE-ɛ4 +ve	*n* (%)	75 (32%)	10 (48%)	18^**b**^ (72%)	**1.46 × 10**^**−4**^
PIB-PET^#^	SUVR (s.d.)	1.425 (0.033)	1.853^**a**^ (0.089)	2.374^**b,c**^ (0.086)	**3.224 × 10**^**−20**^
MRI hippocampus (right, cm^3^)^#^	Mean (s.d.)	3.102 (0.030)	2.970 (0.106)	2.858^**b**^ (0.107)	**0.008**
MRI hippocampus (left, cm^3^)^#^	Mean (s.d.)	3.200 (0.031)	3.142 (0.108)	3.075^**b**^ (0.109)	**3.600 × 10**^**−5**^
MRI gray matter (cm^3^)^#^	Mean (s.d.)	671 (4.611)	694 (16.207)	633^**b,c**^ (16.316)	0.029
MRI white matter^#^ (cm^3^)	Mean (s.d.)	437 (4.514)	438 (15.866)	449 (15.973)	0.660
MMSE	Mean (s.d.)	28.592 (0.119)	27.027^**a**^ (0.388)	20.686^**b,c**^ (0.382)	**9.453 × 10**^**−54**^
CDR scores^#^	Mean (s.d.)	0.027 (0.013)	0.493^**a**^ (0.036)	0.827^**b,c**^ (0.035)	**9.139 × 10**^**−56**^
CDR sum of boxes^#^	Mean (s.d.)	0.039 (0.045)	1.325^**a**^ (0.134)	4.163^**b,c**^ (0.125)	**3.384 × 10**^**−73**^

ANCOVA models adjusted for age, sex, and APOEε4 genotype. The mean and standard deviation (s.d.) are presented in the table with false discovery rate correction set at 5%

^a^CN vs MCI

^b^CN vs AD

^c^MCI vs AD

^#^AIBL group only

Of the plasma proteins implicated in iron and heme metabolism, Hbβ subunit (*p* = 0.001) was significantly increased at baseline in AD compared to CN individuals (Table 2).

**Table 2:** Baseline levels of plasma proteins involved in iron and heme metabolism in CN, MCI, and AD individuals

Plasma proteins		CN	MCI	AD	*p* value
Ceruloplasmin	Mean (s.d.)	− 0.001 (0.004)	0.002 (0.013)	0.003 (0.012)	0.867
Transferrin	Mean (s.d.)	0.000 (0.006)	− 0.008 (0.019)	0.003 (0.0170	0.698
Haptoglobin	Mean (s.d.)	0.003 (0.007)	− 0.025 (0.025)	− 0.007 (0.023)	0.551
Haptoglobin-related protein	Mean (s.d.)	0.002 (0.008)	− 0.019 (0.027)	− 0.007 (0.024)	0.787
Hemopexin	Mean (s.d.)	− 0.003 (0.006)	− 0.016 (0.020)	0.037 (0.018)	0.059
Hemoglobin alpha 1 subunit	Mean (s.d.)	− 0.009 (0.012)	− 0.019 (0.039)	0.078 (0.036)	0.103
Hemoglobin beta subunit	Mean (s.d.)	− 0.017 (0.012)	0.019 (0.041)	0.116^b^ (0.038)	**0.001**
Hemoglobin delta subunit	Mean (s.d.)	− 0.013 (0.012)	0.021 (0.040)	0.086^b^ (0.037)	0.036
CD163	Mean (s.d.)	0.004 (0.01)	− 0.002 (0.034)	0.007 (0.031)	0.886
LRP1	Mean (s.d.)	0.008 (0.019)	− 0.077 (0.064)	− 0.008 (0.059)	0.424

ANCOVA models adjusted for age, sex, and APOEε4 genotype. The mean and standard deviation (s.d.) are presented in the table with false discovery rate correction set at 5%

Units of measurement = a.u

^a^CN vs MCI

^b^CN vs AD

^c^MCI vs AD

### Relationship between baseline plasma proteins and neuroimaging/cognitive measures in CN, MCI, and AD

**Fig. 1:**
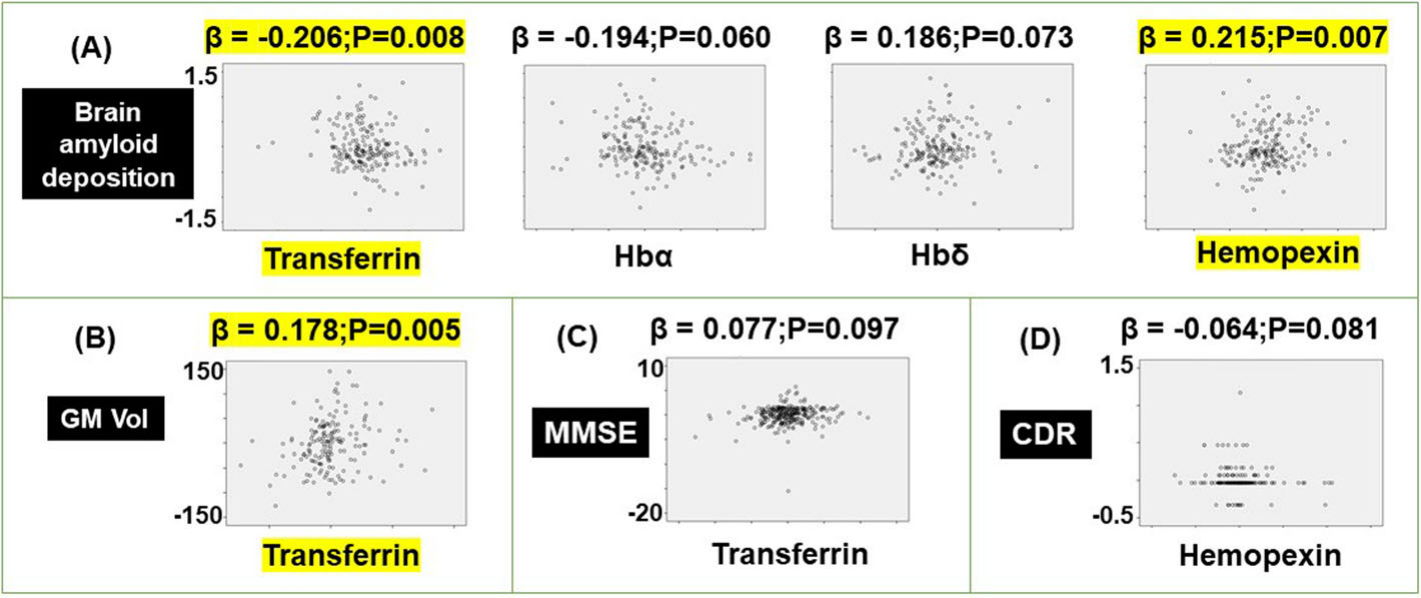
Modeling the association of plasma ceruloplasmin, transferrin, hemopexin, hemoglobin (Hb) α1, Hbβ, Hbδ, haptoglobin, haptoglobin-related protein (HPR), cluster of differentiation (CD) 163, and low-density lipoprotein receptor-related protein 1 (LRP1) with **a** brain amyloid deposition, **b** gray matter volume (GM Vol), **c** mini-mental state examination (MMSE), and **d** clinical dementia rating scale (CDR) score, in cognitively normal, mild cognitive impairment, and Alzheimer’s disease, in the AIBL cohort. The models included age, sex, APOEε4 genotype, and diagnosis as covariates. The standardized coefficient (*β*) and *p* values were stated: significant values that survive the false discovery rate correction set at 5% are highlighted in yellow

Multiple regression modeling of brain amyloid deposition (assessed by PET) revealed lower levels of transferrin (*β* = − 0.206, *p* = 0.008) and a trend of lower Hbα (*β* = − 0.194, *p* = 0.060, not significant), but higher HPX levels (*β* = 0.215, *p* = 0.007) and a trend of increased Hbδ (*β* = 0.186, *p* = 0.073, not significant) were associated with augmented brain amyloid deposition (Fig. 1a). Higher levels of transferrin were associated with larger gray matter volume (*β* = 0.178, *p* = 0.005; Fig. 1b) and non-significant improvements in MMSE scores (*β* = 0.077, *p* = 0.097; Fig. 1c). Higher levels of hemopexin were associated with lower CDR scores that failed to reach significance (*β* = − 0.064, *p* = 0.081; Fig. 1d).

### Baseline levels of brain amyloid deposition, cognition, and plasma proteins in CN individuals

CN individuals from the AIBL cohort stratified according to their conversion status (Table 3) demonstrated elevated brain amyloid deposition in individuals exhibiting progression to cognitive impairment compared to stable CN subjects (*p* = 0.008), while MMSE scores were lower in the former (*p* = 0.006). No significant alterations in the plasma iron or heme proteins were observed in individuals remaining stable or showing disease progression (Table 4).

**Table 3:** Baseline demographic and clinical characteristics of CN individuals remaining stable and individuals showing progression to cognitive impairment (AIBL cohort)

		Stable	Progression	*p* value
*n*	–	115	23	NA
Age	Years (s.d.)	72 (0.670)	72 (1.539)	0.761
Female	*n* (%)	61 (53%)	9 (39%)	0.407
APOE-ɛ4 +ve	*n* (%)	39 (34%)	13 (57%)	0.044
Brain amyloid deposition	PET SUVR (s.d.)	1.35 (0.03)	1.62 (0.08)	**0.008**
MRI hippocampus (right, cm^3^)	Mean (s.d.)	3.12 (0.03)	3.08 (0.07)	0.823
MRI hippocampus (left, cm^3^)	Mean (s.d.)	3.22 (0.03)	3.17 (0.06)	0.642
MRI gray matter (cm^3^)	Mean (s.d.)	674 (4.69)	668 (10.69)	0.633
MRI white matter (cm^3^)	Mean (s.d.)	436 (5.099)	441 (11.049)	0.666
MMSE	Mean (s.d.)	28.895 (0.114)	28.127 (0.248)	**0.006**
CDR scores	Mean (s.d.)	0.024 (0.010)	0.021 (0.022)	0.893
CDR sum of boxes	Mean (s.d.)	0.035 (0.017)	0.020 (0.037)	0.719

ANCOVA models adjusted for age, sex, and APOEε4 genotype. The mean and standard deviation (s.d.) are presented in the table with false discovery rate correction set at 5%

**Table 4:** Baseline levels of plasma proteins implicated in iron and heme metabolism in CN individuals remaining stable and individuals showing progression to cognitive impairment (AIBL cohort)

Plasma proteins	Units	Stable	Progression	*p* value
Ceruloplasmin	Mean (s.d.)	0.001 (0.006)	0.001 (0.014)	0.867
Transferrin	Mean (s.d.)	0.003 (0.008)	0.007 (0.019)	0.794
Haptoglobin	Mean (s.d.)	0.005 (0.01)	0.025 (0.024)	0.458
Haptoglobin-related protein	Mean (s.d.)	0.003 (0.011)	0.036 (0.025)	0.216
Hemopexin	Mean (s.d.)	0.000 (0.010)	− 0.012 (0.022)	0.844
Hemoglobin alpha 1 subunit	Mean (s.d.)	− 0.014 (0.016)	0.042 (0.036)	0.067
Hemoglobin beta subunit	Mean (s.d.)	− 0.024 (0.016)	0.015 (0.037)	0.263
Hemoglobin delta subunit	Mean (s.d.)	− 0.020 (0.016)	0.024 (0.037)	0.204
CD163	Mean (s.d.)	0.009 (0.015)	− 0.012 (0.035)	0.465
LRP1	Mean (s.d.)	0.007 (0.026)	0.028 (0.060)	0.901

ANCOVA models adjusted for age, sex, and APOEε4 genotype. The mean and standard deviation (s.d.) are presented in the table with false discovery rate correction set at 5%

Units of measurement = a.u

### Relationship between plasma proteins and neuroimaging/cognitive measures in CN individuals

**Fig. 2:**
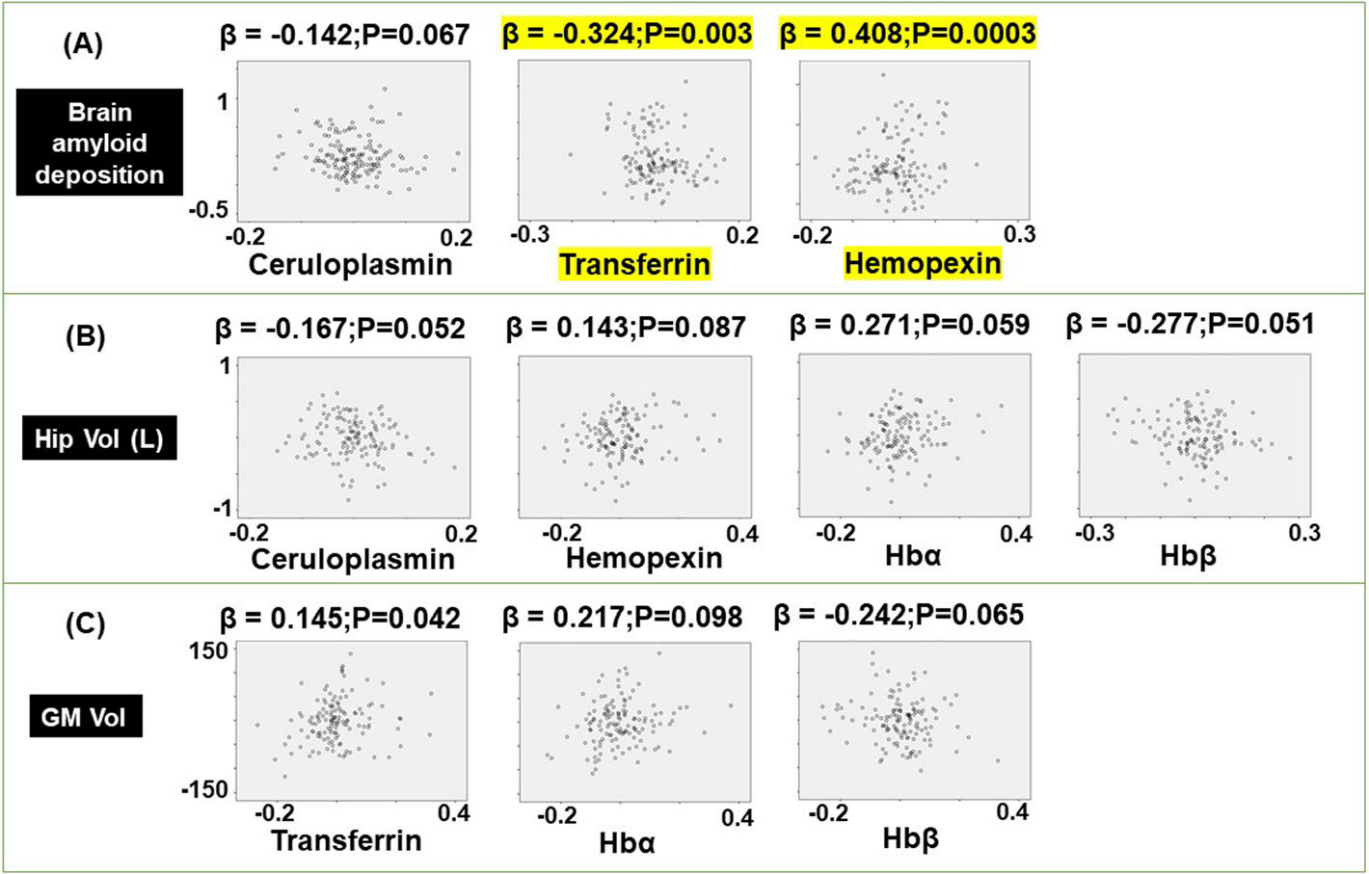
Modeling the association of plasma ceruloplasmin, transferrin, hemopexin, hemoglobin (Hb) α1, Hbβ, Hbδ, haptoglobin, haptoglobin-related protein (HPR), cluster of differentiation (CD) 163, and low-density lipoprotein receptor-related protein 1 (LRP1) with **a** brain amyloid deposition, **b** left hippocampal volume (Hip Vol (L)), and **c** gray matter volume (GM Vol) in the AIBL cohort. The models included age, sex, APOEε4 genotype, and conversion status as covariates. The standardized coefficient (*β*) and *p* values were stated: significant values that survive the false discovery rate correction set at 5% are highlighted in yellow

**Fig. 3:**
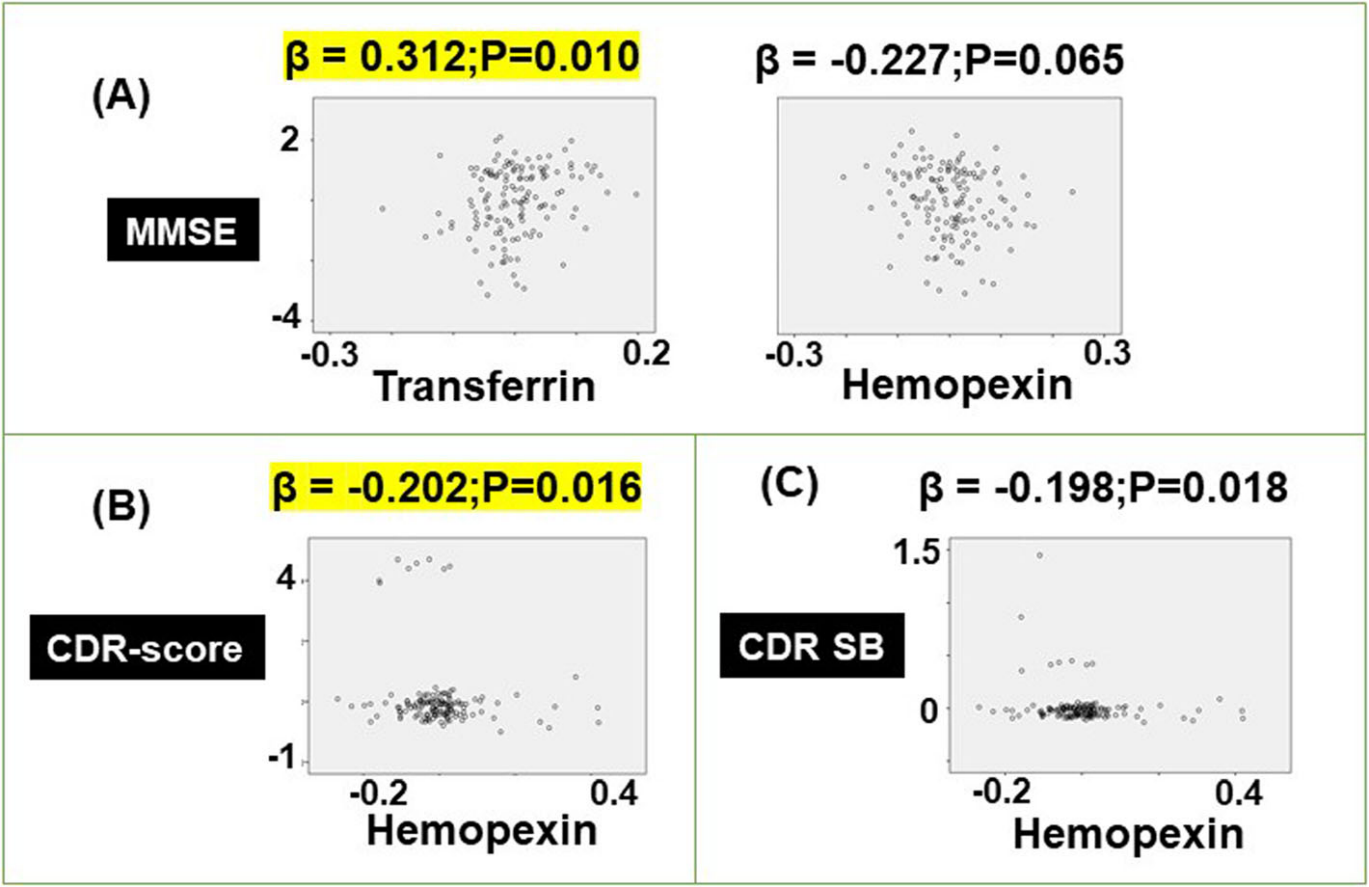
Modeling the association of plasma ceruloplasmin, transferrin, hemopexin, hemoglobin (Hb) α1, Hbβ, Hbδ, haptoglobin, haptoglobin-related protein (HPR), cluster of differentiation (CD) 163, and low-density lipoprotein receptor-related protein 1 (LRP1) with **a** mini-mental state examination (MMSE), **b** clinical dementia rating scale (CDR) score, and **c** clinical dementia rating sum of boxes (CDR SB), in the AIBL cohort. The models included age, sex, APOEε4 genotype, and diagnosis as covariates. The standardized coefficient (*β*) and *p* values were stated: significant values that survive the false discovery rate correction set at 5% are highlighted in yellow

Multiple regression modeling of brain amyloid deposition in CN subjects demonstrated lower levels of transferrin (*β* = − 0.324, *p* = 0.0003) and ceruloplasmin (*β* = − 0.142, *p* = 0.067), but higher levels of HPX (*β* = 0.408, *p* = 3.34 × 10^−4^) were associated with increased amyloid deposition (Fig. 2a). The left hippocampal volume was negatively correlated to ceruloplasmin (*β* = − 0.167, *p* = 0.052) and Hbβ (*β* = − 0.277, *p* = 0.051) but positively correlated to hemopexin (*β* = 0.143, *p* = 0.087) and Hbα (*β* = 0.271, *p* = 0.059), but failed to reach significance (Fig. 2b). Meanwhile, higher levels of transferrin (*β* = 0.145, *p* = 0.042) and Hbα (*β* = 0.217, *p* = 0.098) but lower levels of Hbβ (*β* = − 0.242, *p* = 0.065) were associated with a larger gray matter volume (Fig. 2c), which did not yield significance. Linear regression modeling with MMSE score as the dependent variable in CN subjects revealed augmented levels of transferrin (*β* = 0.312, *p* = 0.010) associated with better performance on the MMSE (Fig. 3a). Lastly, hemopexin was negatively correlated to the CDR score (*β* = − 0.202, *p* = 0.016; Fig. 3b) and CDR sum of boxes (*β* = − 0.198, *p* = 0.018; Fig. 3c). However, the latter did not pass the FDR-corrected *p* value threshold for significance.

### Association between heme and iron proteins in CN stable and progression groups

**Fig. 4:**
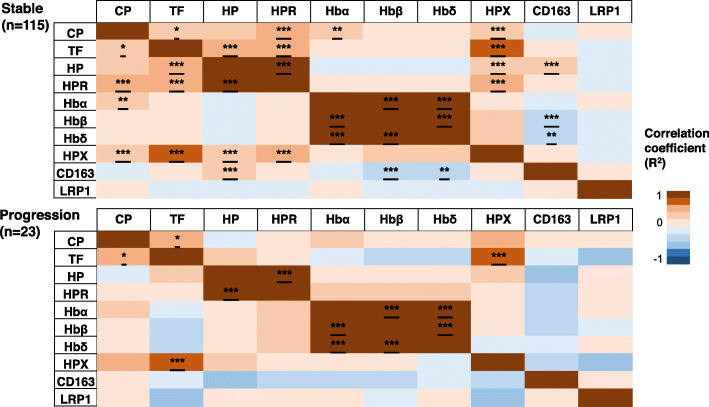
Partial correlation analysis adjusted for age, sex, and APOEε4 genotype conducted separately in cognitively normal AIBL individuals remaining stable and showing progression to cognitive impairment. The scale bar represents the correlation coefficient (*R*^2^) and false discovery rate-corrected significance set at 5% represented by *p < 0.015, **p < 0.009, and *p* < 0.005***. Cp, ceruloplasmin; TF, transferrin; HP, haptoglobin; HPR, haptoglobin-related protein; Hb, hemoglobin subunits (α, β, δ); HPX, hemopexin; CD163, cluster of differentiation 163; LRP1, low-density lipoprotein receptor-related protein

To explain the differences in association between heme and iron proteins, partial correlation analysis was performed and illustrated as a heatmap with different association profiles in CN subjects remaining stable and those progressing to become cognitively impaired (Fig. 4). In the stable population, higher levels of ceruloplasmin were associated with elevated levels of HPR (*r* = 0.422, *p* = 3.523 × 10^−6^), Hbα (*r* = 0.245, *p* = 0.008), Hbβ (*r* = 0.191, *p* = 0.044, did not reach FDR-corrected significance), and hemopexin (*r* = 0.380, *p* = 3.502 × 10^−5^), but this pattern of association was absent in the progression group. Also, ceruloplasmin was positively associated with haptoglobin in the stable group (*r* = 0.222, *p* = 0.019) but did not pass the FDR-corrected significance threshold. Further, transferrin was positively associated with haptoglobin (*r* = 0.454, *p* = 4.939 × 10^−7^) and HPR (*r* = 0.467, *p* = 2.156 × 10^−7^) in the stable group but not significant in the progression cohort. In the progression group, transferrin was negatively correlated to Hbδ but did not reach significance (*r* = − 0.440, *p* = 0.052).

Further associations were observed in CN individuals with stable cognition including higher haptoglobin levels which were associated with augmented levels of hemopexin (*r* = 0.402, *p* = 1.108 × 10^−5^) and CD163 (*r* = 0.321, *p* = 5.565 × 10^−4^). On the contrary, CN subjects showing progression to cognitive impairment showed negative correlation between haptoglobin and CD163 levels (*r* = − 0.437, *p* = 0.054) but did not reach significance. Meanwhile, HPR was positively correlated to the hemopexin (*r* = 0.539, *p* = 8.389 × 10^−10^) only in the stable population. Both the Hbβ and Hbδ subunits were positively associated with hemopexin (*r* = 0.205, *p* = 0.030; *r* = 0.208, *p* = 0.028 respectively) but did not surpass the FDR-corrected significance threshold. Also, Hbβ and Hbδ subunits were negatively associated with CD163 only in the stable group (*r* = − 0.284, *p* = 0.002; *r* = − 0.257, *p* = 0.006). Hbα was associated with Hbβ and Hbδ in the stable (*r* = 0.841, *p* = 3.643 × 10^−31^; *r* = 0.839, *p* = 6.994 × 10^−31^) and progression groups (*r* = 0.835, *p* = 4.701 × 10^−6^; *r* = 0.811, *p* = 0.141 × 10^−5^). Similarly, Hbβ demonstrated significant correlation to Hbδ in the stable group (*r* = 0.946, 1.218 × 10^−55^) and progression group (*r* = 0.885, *p* = 2.196 × 10^−7^).

## Discussion

AD causes a global disease burden involving failure of neuroprotective mechanisms leading to altered heme/iron dyshomeostasis and oxidative stress-mediated neurodegeneration [47]. In keeping with the theme of heme dyshomeostasis [48], in this study, we found significantly increased plasma levels of Hbβ subunit in AD compared to CN individuals. A trend was apparent where lower Hbα and higher levels of Hbδ were associated with increased brain amyloid deposition. How Hb or its metabolites pass the blood-brain barrier remains to be fully understood. It has been posited that increased permeability of the blood-brain barrier early in the disease process may increase Hb concentration in the brain vasculature and explain the higher prevalence of cerebral amyloid angiopathy in AD [49, 50]. Further, increased Hb in the brains of human and transgenic mouse models of AD has been suggested to induce astrocytic fatigue, enabling pathogenesis mediated by Aβ [48, 49]. This is in congruence with our finding of a trend of lower plasma levels of Hbα associated with increased brain amyloid deposition.

**Fig. 5:**
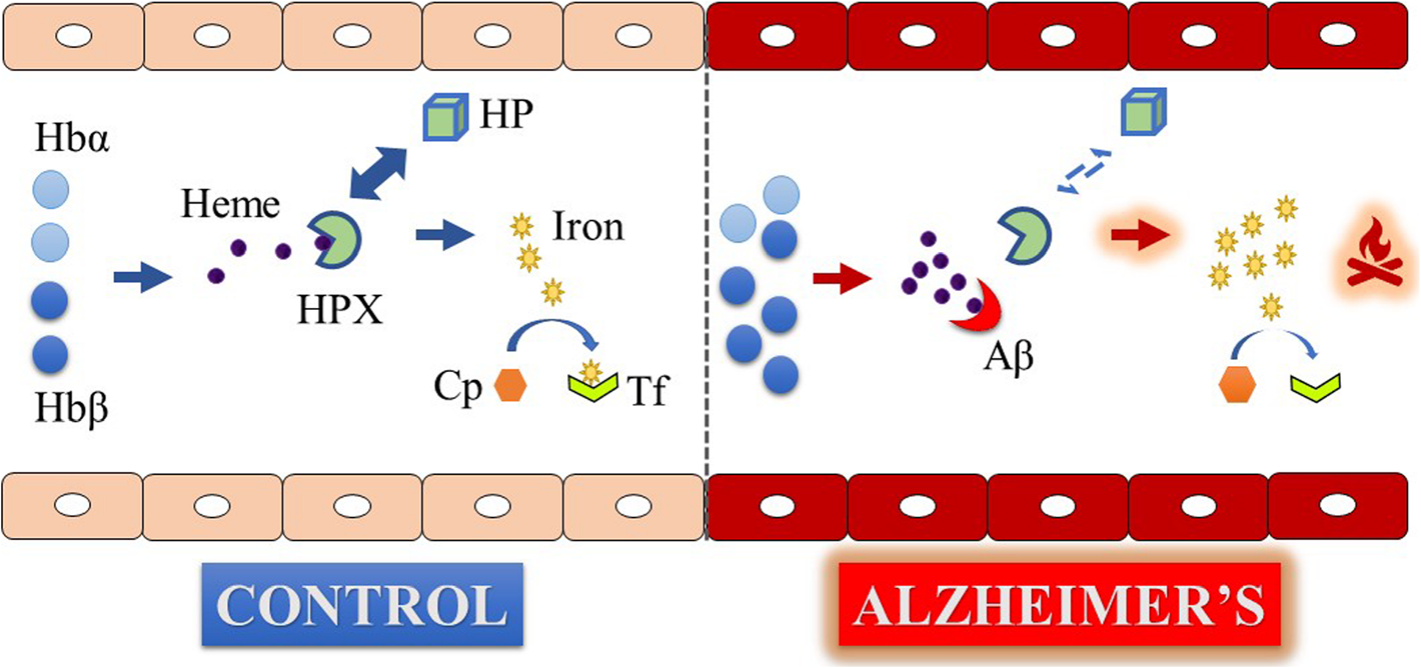
Schematic to summarize the main findings of the study. Hemoglobin (Hb) α and β derived heme is scavenged by hemopexin (HPX) complemented by its co-partner haptoglobin (HP) to form HPX-heme complex. Heme contributes to redox-active iron which is loaded onto transferrin (Tf) with the help of ceruloplasmin (Cp) to maintain iron homeostasis. In the presence of increasing Alzheimer’s pathology, augmented levels of Hbβ leads to excess free heme, which in the presence of a dissociation between HPX and HP impairs the heme detoxifying system. This enables β-amyloid (Aβ) to readily form complexes with heme to increase the redox-active iron pool. Since higher levels of Aβ were associated with lower levels of Tf, the ability of Tf to circulate iron is compromised. The impaired homeostasis of iron leads to enhanced oxidative stress, which contributes to neurodegeneration and progression of Alzheimer’s pathology

Blood from healthy aged humans (29–51 years) and 8-week old young adult mice showed that Hbα subunits can efficiently remove hydrogen peroxide to prevent oxidative degradation of Hb, and its ability to scavenge is overtly enhanced in the presence of reduced pyridine nucleotides [20]. Previously, it has been documented that Hb and heme may help to suppress Aβ-mediated inflammatory activation of astrocytes [49]. Although we report correlation, not causation, the varying association between different subunits leads us to hypothesize that Hb subunits may modify Aβ-mediated toxicity and ameliorate oxidative stress, probably in divergent ways. This is supported by our finding of an increasing trend of Hbα associated with increased hippocampal volume in CN individuals (adjusted for conversion status), but the relationship was found to be the converse for Hbβ, suggesting perturbations in heme biology is an early event in AD pathogenesis (Fig. 5).

Interestingly, our data showed that increased brain amyloid deposition was associated with higher levels of plasma HPX in the whole cohort, but higher HPX was associated with a lower CDR score in CN individuals (adjusted for conversion status). HPX co-partners with haptoglobin to bind heme with high affinity and confers protection against free heme-mediated toxicity, maintaining iron homeostasis via recycling of heme iron [28]. HPX knockout mice studies have further substantiated its role as an effective heme/iron scavenger [32]. We observed a significant positive association between HPX and haptoglobin and a trend for Hbβ and Hbδ, only in CN individuals who remained stable, but this association was absent in those that progressed to cognitive impairment. This suggests that under normal conditions, HPX scavenges and detoxifies heme, but under pathological conditions such as in AD, Aβ disrupts HPX binding to heme through formation of Aβ-heme complex, thereby promoting peroxidase activity and accentuating oxidative stress [51–54]. The peroxidase activity of Aβ-heme complexes have been shown to induce peptide dimer formation, which in turn enhance Aβ fibrillization [55]. The formation of Aβ-heme complex can decrease heme bioavailability of heme, in effect, inducing functional heme deficiency [51]. The excessive build-up of heme derived from Hb has been shown to aggravate tau aggregation via the N-terminal free-amines, prolonging peroxidase activity and perpetuating neuronal oxidative stress [56].

Unexpectedly, increased HPX levels correlated with a trend of a decrease in MMSE. However, elevated HPX levels may not reflect increased function as HPX may well be oxidized/inactivated under pathological conditions operant in AD [57, 58]. The only known HPX-heme complex receptor has been identified as LRP1 (also known as CD91), which is expressed in several cell types including macrophages, hepatocytes, neurons, and syncytiotrophoblasts [59] and is responsible for mediating HPX-heme internalization into cells for heme detoxification. We did not observe a significant association between LRP1 and HPX in the CN stable or progression group. However, the role of LRP1 expressing cell types needs to be elucidated alongside putative contribution of alternative receptors for HPX-heme clearance.

Under physiological conditions, haptoglobin binds avidly to Hb and at macrophages, following binding to its cognate receptor, CD163, the complex is endocytosed, thereby mediating heme and iron homeostasis [28, 60]. In our dataset, higher levels of haptoglobin were associated with increased levels of CD163 in stable CN individuals but demonstrated a trend of negative association in those showing progression to cognitive impairment. This could imply in the latter CN individuals, uptake of the haptoglobin-Hb complex by CD163 is inhibited, allowing aberrant heme signaling to increase redox-active iron, which precipitates oxidative stress in AD. The chaperone, haptoglobin, has also been shown to be susceptible to oxidation which hinders its scavenging ability, and in AD, enabling Aβ to readily bind heme, further exacerbating Aβ-induced toxicity [61, 62].

Consistent with previous studies, we did not find significant alterations in plasma transferrin levels in the CN versus the disease groups. Nevertheless, we demonstrate lower levels of transferrin are associated with augmented brain amyloid uptake. This could particularly be problematic in AD, where deficiency of iron loading into transferrin [7] may enable non-transferrin-bound iron to detrimentally contribute to the labile iron pool, which could help propagate Aβ-toxicity. Our finding of increased transferrin levels being associated with increased hippocampal volumes and improved MMSE scores underscores the importance of the role of transferrin to bind iron in a safe and redox-inactive form to distribute elsewhere. The relationship between plasma and brain iron needs to be established; one could hypothesize that the decreases in plasma iron [7, 9] is due to increased partitioning of iron into the brain in AD [63]. Macrophages have been suggested to require the ferroxidase activity of ceruloplasmin to load iron onto transferrin. We did not find an increase in ceruloplasmin levels, but ceruloplasmin concentration is not a measure of its activity since oxidation of ceruloplasmin can impair its ferroxidase activity [64] and inhibit iron loading onto transferrin in AD [8]. One limitation of the study was that we did not investigate protein activities, which could be a useful corollary study. We performed correlation analysis to evaluate the relationship between heme and iron proteins, which does not necessarily equate to causation; hence, future experiments should be directed at identifying causal relationships. In summary, it would be beneficial to understand the expression levels/activity of heme and iron proteins in the plasma, CSF, and tissues of the same subjects to more fully understand heme and iron distributions between various compartments and elucidate the mechanistic interplay.

Perturbed iron homeostasis is a feature of several neurodegenerative diseases including AD, Parkinson’s disease, frontotemporal dementia, and amyotrophic lateral sclerosis. We did not study the impact of tau which correlates better with disease progression than Aβ [65] or the contribution of α-synuclein, tau, and TDP43 pathologies in the present study, as these pathologies are prevalent in 50% and 30% of AD patients respectively [66]. We predict that these proteins may affect levels of heme and iron proteins, as iron is required for seeding and aggregation of tau [67], of α-synuclein [68], and, indirectly, of TDP43 [69, 70]. We have recently provided evidence to support the presence of a recently discovered iron-dependent cell death, ferroptosis, in AD [71]. Iron chelation has shown promise against AD and PD in preclinical studies and clinical trials and has therapeutic potential to extinguish iron-dependent ferroptosis, a mechanism proposed to contribute to neurodegeneration [67]. With relevance to AD, longitudinal studies have shown iron burden to be associated with pronounced increase in rate of cognitive decline in Aβ-positive individuals [63, 72, 73]. Moreover, heme/iron accumulation is preferentially observed in AD affected regions of the brain including parietal cortex, motor cortex, and hippocampus [19, 74–78]. Interestingly, accumulation of tau neurofibrillary tangles is associated with heme oxygenase-1 induction which may further accentuate oxidative stress via release of redox-active iron from heme catabolism [79–81]. We did not perform [^18^F]FDG-PET to measure glucose metabolism but predict that heme/iron homeostatic proteins would be closely associated with hypometabolism in AD. Whether iron is associated with region-specific effects of neurodegenerative diseases and how it interacts with different pathological proteins/aggregates would be an exciting avenue to explore.

Importantly, heme is derived from Hb, which is enriched in red blood cells [15]. In AD, erythrocytes have been found to contain more surface-bound IgG displaying augmented proteolysis and ensuing oxidative stress, which may trigger their lysis and a subsequent increase in free Hb fragments [82, 83]. Together with the reduced heme scavenging potential apparent in AD, this may in part explain our finding of increased plasma Hbβ in AD and the varying associations we observed. However, longitudinal studies are warranted to simultaneously temporally stage CSF Hb during the progression of AD to understand brain-periphery relationships. Detailed mechanistic studies are required to understand post-translational modifications of Hb and their associations with Aβ and tau. Also, different neuronal cell lineages can be transfected to upregulate or downregulate Hbα and Hbβ, to test their vulnerability to Aβ and tau toxicity. In transgenic mouse models of AD, cell type-specific (neuron, microglia, and astrocytes) deletions and overexpression of Hb chains can be performed to assess whether these modifications will alleviate or aggravate the disease burden.

## Conclusion

In conclusion, heme (iron) dyshomeostasis appears to be a feature of AD. The causal relationship between heme/iron metabolism and AD warrants further investigation.

## Data Availability

All data needed to evaluate the conclusions in the paper are present in the paper. Additional data related to this paper may be requested from the authors.

## Abbreviations

AD: Alzheimer’s disease
AIBL: Australian Imaging, Biomarker and Lifestyle Flagship Study of Ageing
CDRSB: Clinical dementia rating scale (CDR) and sum of boxes
CD163: Cluster of differentiation 163
CN: Cognitively normal
TE: Echo time
FSE: Fast spin echo
18F-FBB: Florbetaben
FLAIR: Fluid attenuation inversion recovery
HPR: Haptoglobin-related protein
Hb: Hemoglobin
HPX: Hemopexin
TI: Inversion time
KARVIAH: Kerr Anglican Retirement Village Initiative in Ageing Health
LRP1: Low-density lipoprotein receptor-related protein
MCI: Mild cognitive impairment
MMSE: Mini-mental state examination
TR: Repetition time
TMT: Tandem mass tag
